# Soft and Hard Textured Wheat Differ in Starch Properties as Indicated by Trimodal Distribution, Morphology, Thermal and Crystalline Properties

**DOI:** 10.1371/journal.pone.0147622

**Published:** 2016-01-29

**Authors:** Rohit Kumar, Aman Kumar, Nand Kishor Sharma, Navneet Kaur, Venkatesh Chunduri, Meenakshi Chawla, Saloni Sharma, Kashmir Singh, Monika Garg

**Affiliations:** 1 National Agri-Food Biotechnology Institute, Mohali, Punjab, India; 2 Panjab University, Chandigarh, Punjab, India; Murdoch University, AUSTRALIA

## Abstract

Starch and proteins are major components in the wheat endosperm that affect its end product quality. Between the two textural classes of wheat i.e. hard and soft, starch granules are loosely bound with the lipids and proteins in soft wheat due to higher expression of interfering grain softness proteins. It might have impact on starch granules properties. In this work for the first time the physiochemical and structural properties of different sized starch granules (A-, B- and C-granules) were studied to understand the differences in starches with respect to soft and hard wheat. A-, B- and C-type granules were separated with >95% purity. Average number and proportion of A-, B-, and C-type granules was 18%, 56%, 26% and 76%, 19%, 5% respectively. All had symmetrical birefringence pattern with varied intensity. All displayed typical A-type crystallites. A-type granules also showed V-type crystallinity that is indicative of starch complexes with lipids and proteins. Granules differing in gelatinization temperature (ΔH) and transition temperature (ΔT), showed different enthalpy changes during heating. Substitution analysis indicated differences in relative substitution pattern of different starch granules. Birefringence, percentage crystallinity, transmittance, gelatinization enthalpy and substitution decreased in order of A>B>C being higher in hard wheat than soft wheat. Amylose content decreased in order of A>B>C being higher in soft wheat than hard wheat. Reconstitution experiment showed that starch properties could be manipulated by changing the composition of starch granules. Addition of A-granules to total starch significantly affected its thermal properties. Effect of A-granule addition was higher than B- and C-granules. Transmittance of the starch granules paste showed that starch granules of hard wheat formed clear paste. These results suggested that in addition to differences in protein concentration, hard and soft wheat lines have differences in starch composition also.

## Introduction

Wheat is one of the most important cereals for direct human consumption. Its textural properties determine the end product quality. Wheat has two textural classes i.e. soft and hard. Two classes of wheat are utilized for different purposes. Hard wheat is mainly used for bread making, pasta making while soft wheat is utilized for biscuit and cake making. The differences in grain hardness arise due to presence or absence of two puroindoline proteins PINA and PINB [1]. The presence of both proteins is associated with soft texture while absence or mutation in any one of them leads to hard texture. Since puroindolines are starch granule bound proteins and involved in grain texture, they might affect starch composition and its properties. During grain development, starch is deposited in the endosperm as discrete semicrystalline aggregates known as starch granules [2]. Wheat has unique trimodal distribution of starch granules i.e. A- (diameter 10–50 μm), B- (diameter 5–9.9 μm) and C-granules (diameter <5 μm) [3,4]. These granules are composed of two types of glucose polymers i.e. amylose and amylopectin. Amylose is a linear molecule of α-1,4-linked glucose residues and having few α-1,6-glycosidic branches [5]. Amylopectin molecules, on the other hand, are much larger (up to millions of residues) and highly branched with a high frequency of α-1,6-glycosidic linkages [6]. The branching of the glucan chains of amylopectin occurs with regular periodicity [7] and its length and pattern are critical for the proper formation and properties of the starch granule. These two kinds of polymers form amorphous and crystalline regions in starch granules [7] that are responsible for their characteristic birefringence pattern and crystallinity. Four types of crystalline structures have been documented [8,9]. The A-type crystalline structure is present in cereal starches such as wheat and rice starches, and the B-type crystalline structure present in tuber, fruit and stem starches such as potato and banana starches. C-type crystalline structure is combination of both A- and B-type structures [10,11]. In V-type crystalline structure, amylose form complexes with compounds such as iodine, dimethyl sulfoxide (DMSO), alcohols, or fatty acids [12]. Starch crystallinity affects thermal properties of the starch granules. Starch with high crystallinity exhibit high enthalpy and require higher energy inputs during gelatinization. Physiochemical properties of each type of starch granule vary and contribute to the food and industrial end-uses of starch [13,14]. No study has been reported on the detailed comparison of the molecular structures of the A-, B-, and C-type starch granules from wheat varying in textural properties. In this study we have investigated soft and hard wheat lines for trimodal starch granules distribution, their granular morphology, crystalline structure and thermal properties.

## Materials and Methods

### Materials

Two wheat genotype, a Indian wheat cultivar (*Triticum aestivum* cv. C306) obtained from Directorate of Wheat Research, Karnal, India (340 m above sea level; Latitude 29° 41’ North, Longitude 76° 58’ East) and a land race (*T*. *aestivum* L. IITR67) obtained from Prof. H.S. Dhaliwal, Indian Institute of Technology, Roorkee and collected from Hilly region of Uttarakhand, India (Latitude 28° 43’ to 31° 27’ North, Longitude 77° 34’ to 81° 02’ East). These two genotypes with contrasting hardness were grown in the experimental field of National Agri-Food Biotechnology Institute (NABI), SAS Nagar (Mohali) Punjab, India (310 m above sea level; Latitude 30° 47’ North, Longitude 76° 41’ East). The harvested seed at maturity were used for starch isolation.

### Grain Hardness

Hardness of mature seeds was determined using Single kernel characterization system (SKCS 4100, Perten Instruments North America Inc., Springfield, IL, USA).

### Starch Granules Isolation

Wheat starch granules were isolated following the method of Peng et al. [15]. Briefly wheat grains (6 g) were steeped in 50 mL of water at 4°C for 16 h. The softened seeds were grinded. The slurry was filtered through four layers of cheesecloth to remove endosperm cell debris and centrifuged at 3500 X g for 5 min. The yellow gel-like layer on top of the packed white starch granule pellet was carefully removed. The starch granule pellet was then suspended in 5 mL of MQ water, overlaid on 30 mL of 80% (w/v) cesium chloride and centrifuged at 3500 X g for 5 min. The starch granule pellet, referred to as the whole-starch granule population, was washed twice with 30 mL of wash buffer (62.5 mM tris-HCl, pH 6.8; 10 mM sodium EDTA; and 4% SDS), four times with water, once with acetone, air dried and stored at -20°C.

### Starch granules fractionation

Sedimentation was used to separate total starch into three fractions A-, B- and C-granules as described by Takeda et al. [16] with modifications. The starch was suspended in water (10 g/ 250 mL) in 500 mL measuring cylinder and allowed to separate under gravity for 2 h. The granules settled at the bottom of measuring cylinder were considered as A-granules. Supernatant consisted of a mixture of B- and C-granules. It was kept for 10 h in another measuring cylinder and upper fraction was collected as C-granules. The fraction settled at the bottom of measuring cylinder was considered as B-granules. All the fractions were collected after centrifugation at 3500 X g for 5 min, washed with acetone, air dried and stored at -20°C.

### Light and polarized microscopy

The size of starch granules i.e. A-, B-, C-granules and total starch (T-type) granules were analyzed by light microscopy. Starch granules (4 mg) were suspended in 200 μl of 90% ethanol. A drop of starch suspension was spread on a microscope slide and air-dried. The slide was then placed on the stage of a light microscope (10 X/.30, Leica DM6000B, Wetzlar, Germany) and analyzed for granule size, shape and birefringence (using polarized light mode). Number of different type of starch granules from total starch was counted using microscopic counter (Sigma-Aldrich, St. Louis, USA) after staining with potassium iodide (KI).

### Scanning electron microscopy (SEM)

Scanning electron microscopy (Variable Pressure Scanning Electron Microscope, Hitachi S3400N, Japan) was used to assess the shape, size and purity of different starch granules. Starch samples (~1 mg) were applied on an aluminum stub using double-sided adhesive tape, and the starch was coated with gold. An acceleration potential of 10.0 kV was used during microscopy at 30X.

### Gel permeation chromatography

10 mg of starch sample was dissolved in 2 ml of DMSO in a boiling water bath. After passing through the Sepharose CL-2B column (GE Healthcare Life Science, California, USA), amylose and amylopectin were separated based on their molecular weight. Fractions were assayed using phenol-sulphuric acid method [17]. The chromatogram revealed two peaks of elution. Amylose content was estimated as a percentage of the total amount of starch within the second peak of elution to the total amount of starch in both the peaks.

### X-ray crystallography (XRD)

XRD of starch granules (~20 mg, equilibrated at 100% relative humidity, at 25°C for 24 h) were recorded using diffractometer (D8, Bruker AXS Inc., Germany), Cu KR radiation with a wavelength of 0.154 nm operating at 45 kV and 40 mA. XRD were acquired at 25°C over a 2θ range of 3–40° with a step size of 0.018° and sampling interval of 52 s. Relative crystallinity was estimated from the ratio of the area of peaks to the total area of diffractogram (2θ).

### Differential scanning calorimetry (DSC)

Thermal properties were analyzed using DSC (822, Mettler Toledo, Columbus, OS, USA) equipped with a thermal analysis data station. The analyzer was calibrated using indium and an empty aluminum pan was used as reference. Sample pans (5 mg starch in 15 μl of water) were heated at a rate of 5°C/min from 40 to 110°C and cooled at the same rate to 40°C. The heating and cooling cycles were repeated three times. Onset temperature (To), peak temperature (Tp), conclusion temperature (Tc) and enthalpy (ΔH) were calculated using stare software for thermal analysis (STARe SW 9.01).

### Reconstitution experiment

Starch granules mixtures were prepared by individually adding 10% (w/w) of A-, B- and C-granules extracted from soft wheat to soft wheat total starch. Similarly, isolated granules of hard wheat were added to hard wheat total starch. In addition, hard wheat granules were added to soft wheat and vice versa. Thermal properties were analyzed using DSC.

### Light transmittance (T%) of starch pastes

Starch granules paste (1%) was prepared by dissolving 10 mg of starch granules in 1 ml of water in boiling water bath for 30 min. After cooling for 5 min, percent transmittance (%T) was determined against water blank at 650 nm using spectrophotometer (SpectraMax M^e5^, Molecular Devices, Sunnyvale, California, US).

### Gas chromatography/ Mass spectroscopy (GC/MS)

Starch granules were methylated following the method of Ciucanu and Kerek [18]. Lyophilized granules (2 mg) were dissolved in DMSO (0.2 ml). Freshly prepared NaOH slurry in DMSO (0.5 ml) was added and the reaction mixture was allowed to stand at room temperature for 15 min. Iodomethane (MeI, 0.5 ml) was added, and samples were stirred at room temperature for 30 min. The samples were partitioned between water (2 ml) and chloroform (2 ml). Organic layer was collected, washed with deionized water, and dried. The dried samples were dissolved in methanol. The methylated molecules (200 μg) were hydrolyzed with 2 N TFA, 120°C for 2 h. The hydrolyzed material was reduced with NaBD4 (Sigma-Aldrich, St. Louis, USA) and acetylated with 1:1 acetic anhydride-TFA at 50°C for 20 min to generate partially methylated alditol acetates. Derivatized monosaccharides were analyzed by gas chromatography (Agilent 7000 GC/MS Triple Quad system, Santa Clara, CA, USA). The sample (1 μl) was injected manually onto a DB-5 (30 m X 0.25 mm, 0.25 μm film thickness) column using helium as carrier gas (Inlet temperature 240°C). Following temperature gradient was used for separation: 80°C for 2 min, 80–170°C at 30°C/min, 170–240°C at 4°C/min, 240°C held for 20 min. Samples were ionized by electron impact at 70 eV and source temperature of 230°C. The peaks were detected on the basis of retention time and molecular mass (mass range 30–500 Da). Detected peaks were identified after matching with CCRC database available online. Data analysis was performed using software (Agilent Mass hunter Quailitative Analysis B.05.00).

### Statistical Analysis

Experiments were carried out in three replicates subjected to an analysis of variance (ANOVA) by Duncan’s test (p< 0.05) using SPSS 16.0 software (SPSS, Inc., Chicago, IL, USA).

## Results and Discussion

### Starch granules distribution

**Fig 1:**
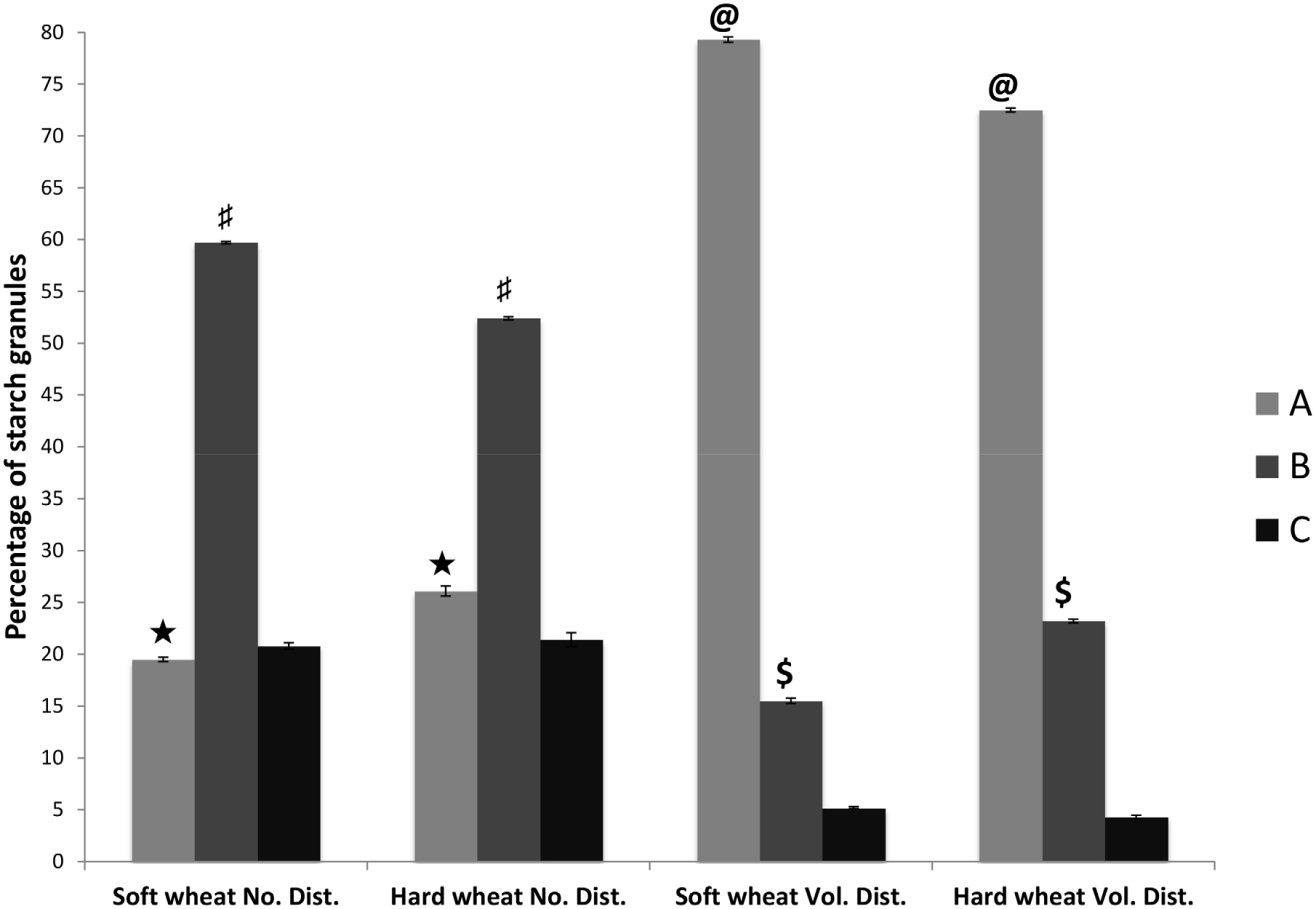
Starch granules number and volume distribution in soft and hard wheat. A, B and C are the A-, B- and C-granules of soft and hard wheat. Graph Bars marked with same sign represent similar type of granules that are significantly different from each other at p value 0.05.

In the present work we were able to separate A-, B- and C-type starch granules with >95% purity. Previous studies have reported morphological data of A- and B-type starch granules [19,20]. In this work we have included C-granules for similar analysis. Our data suggested that B-granules were highest in number compare to A- and C-granules (Fig 1). Our data indicated average number of A-, B- and C-granules as 18%, 56% and 26% respectively. This observation is different from previous studies that have reported total number of A- and B-type starch granules as 10% and 90% respectively [21,22]. Soft wheat had more number of B-granules and hard wheat had more number of A-granules (Fig 1). Numbers of C-granules were non-significantly different between soft and hard wheat. Average proportion of A-, B- and C-granules was 76%, 19% and 5% respectively. Our results are different from previous studies, which reported that A- and B- granules account for 70% and 30% of the starch volume [21,22]. Soft wheat had higher proportion of A- and C-granules and hard wheat had higher proportion of B-granules (Fig 1). But B-granules were higher in number in soft wheat than hard wheat that might be due to smaller size of B-granules in soft wheat compared to hard wheat. The number proportions of B-granules were high in both the genotypes but their contribution to volume was less because of their smaller size [21].

### Size, shape and birefringence of starch granules

**Fig 2:**
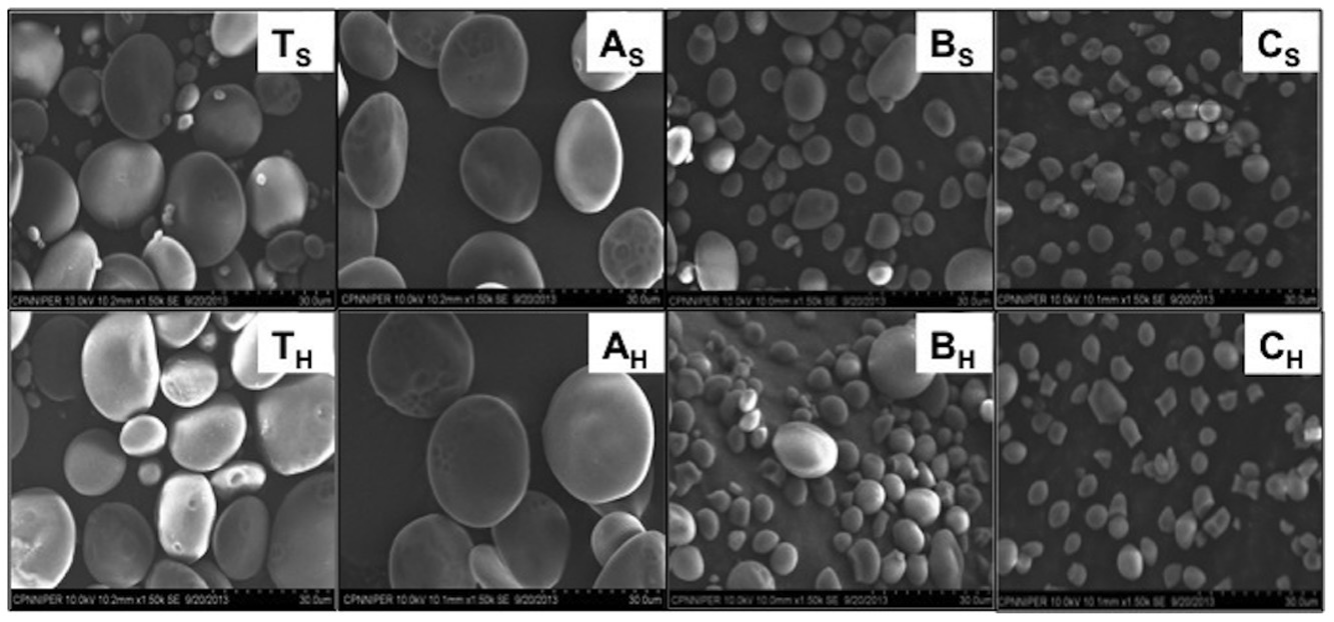
Scanning electron microscopic images of starch granules at 30x. T_S_, A_S_, B_S_ and C_S_ represents soft wheat total starch, A granules, B granules and C granules respectively. T_H_, A_H_, B_H_ and C_H_ represents hard wheat total starch, A granules, B granules and C granules respectively.

Light and scanning electron microscopy of total starch, A-, B-, C-granules from soft and hard wheat indicated significant size variation between different types of granules and non-significant variation within starch granules from different textured wheat (S1 Fig and Fig 2). A-granules displayed a disk or lenticular shape with diameters >10 μm, B-granules displayed a spherical shape with diameters of about 3–10 μm and C-granules displayed a irregular shape with diameter <3 μm as reported previously [3,4]. The different sizes of the starch granules might be attributed to their different time of formation during grain development [23]. A-granules are formed around 4–14 days post anthesis (DPA) when the endosperm is still actively dividing [24,25,26]. B-granules are initiated at about 10–16 DPA in stromules (stroma containing tubules) that are extruded from A-granule containing plastids [22], and the small C-granules first appear about 21 DPA [4]. Irregular shape of C-granules might be due to their small size and tight packing in the seed. Starch granules exhibit orderly arrangement of crystalline region and disorderly arrangement of amorphous region. This anisotropy gives starch granules characteristics birefringence (polarization cross or Maltese cross) pattern that can be observed under plane polarized light [27].

**Fig 3:**
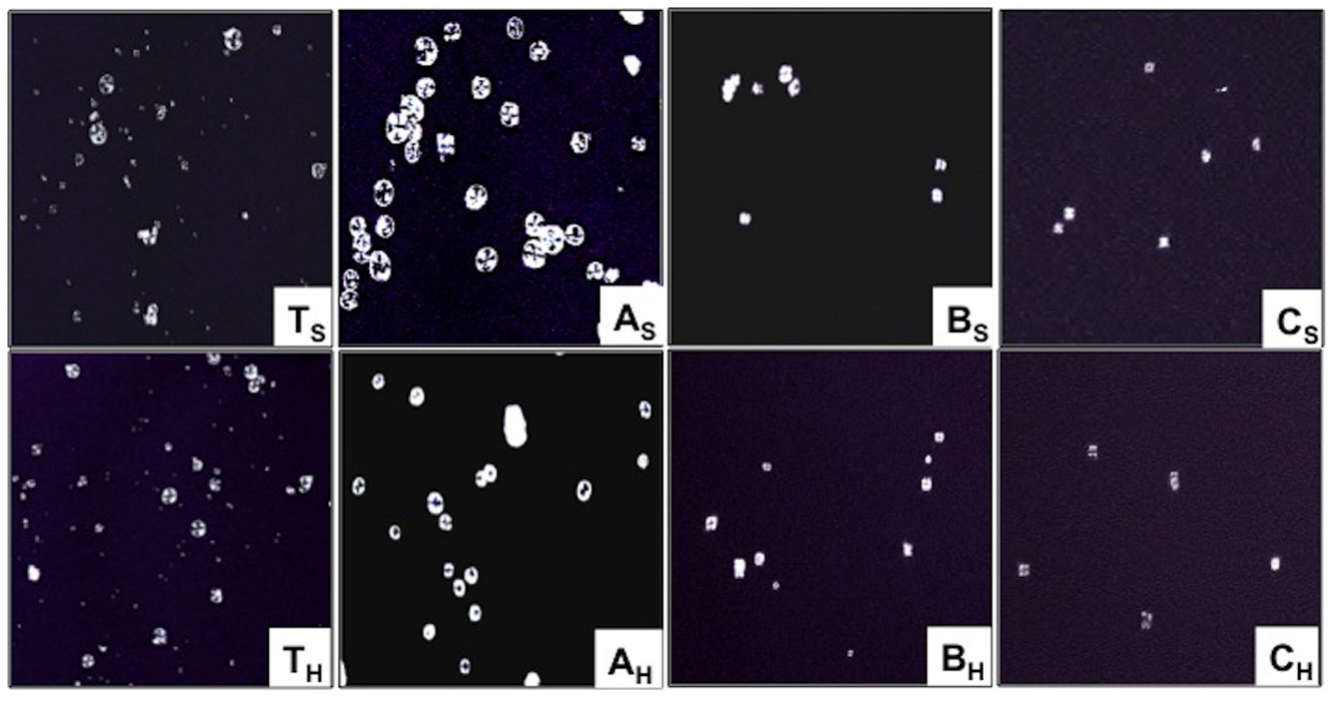
Birefringence images of starch granules.

The intensity of birefringence depends on the granule size, relative crystallinity, and microcrystalline orientation. Polarized microscopy of A-, B- and C-granules indicated that A-granules had higher birefringence intensity than B- and C-type granules (Fig 3). Relatively weak and obscure polarization cross was observed in total starch, B- and C-type granules. Similar observations had been reported for A- and B-type granules [20]. Hard wheat granules showed higher birefringence intensity than soft wheat granules. Higher birefringence in A granules might be due to higher order of crystallite organization than B and C granules. Irregular shape of C might be attributing towards their obscure birefringence.

### Crystallinity of starch granules

**Fig 4:**
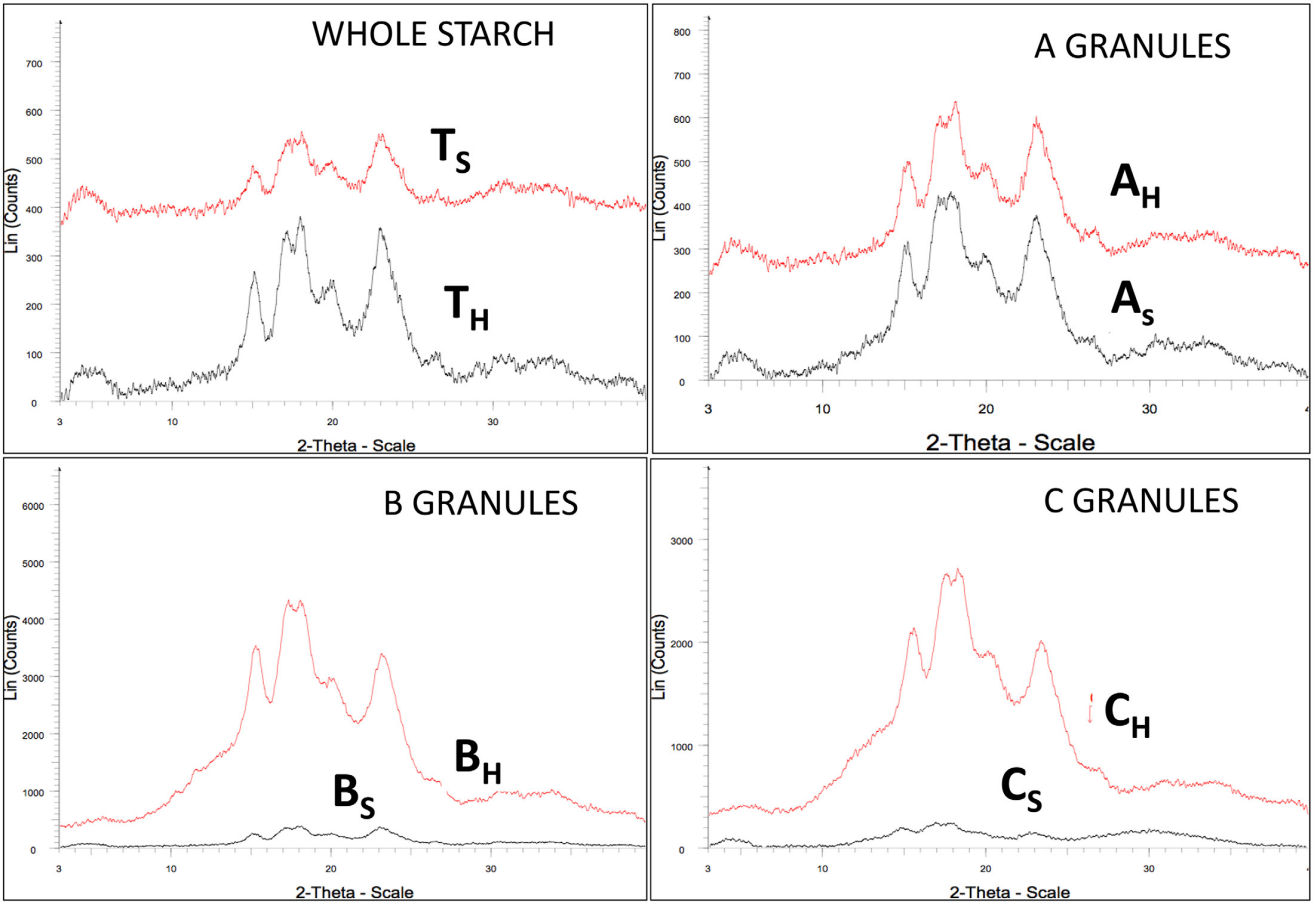
XRD pattern of starch granules.

X-ray diffraction pattern of total starch, A-, B- and C-granules have been shown in Fig 4. All starch granules showed strong diffraction peaks at 15°, 17°, 18° and 23° [20]. These peaks are characteristics for A-type crystallinity. As starch molecules forms crystalline and amorphous region in starch granules therefore a characteristic XRD pattern is obtained for a particular type of starch. In XRD pattern crystalline part shows sharp peaks while amorphous part shows dispersive peaks [10]. Total starch and A granules showed additional diffraction peak at 20°. Additional peaks at 20° is indicative of V-type crystallinity. In V-type crystallinity amylose form complexes with compounds such as iodine, DMSO, alcohols, or fatty acids [12]. In wheat complexes of starch, lipids and proteins are known [28]. Starch granules of both varieties showed differences in peak intensities and relative crystallinity (Table 1). Percentage crystallinity of hard wheat granules was higher than soft wheat granules. Percentage crystallinity decreased in the order of A> B> C. These results are consistent with birefringence results (Fig 3). The differences in the crystallinity of soft wheat starch granules were more prominent than hard wheat. Our results are in agreement with the previous results for A-, B-granules [20] and for the C granules we have characterized for the first time. Higher crystallinity of larger granules might be due to better crystallite formation in them. Although the volume proportion of A granules was more in soft wheat than hard wheat still the crystallinity and birefringence of hard wheat was higher than the soft one. This might be due the higher crystallinity of B and C granules of hard wheat in addition to A-granules.

**Table 1:** Summary of the results for amylose content, Crystallinity and thermal properties for starch granules.

Sample Name	% age Amylose content	% age crystallinity	To (°C)	Tp (°C)	Tc (°C)	ΔT (Tc-To)	ΔH (J/g)
Soft wheat whole starch	25.78±0.34f	26.13±0.06^d^	56.3±0.34a	60.51±0.17a	66.13±0.06b	9.83±0.33c	8.23±0.052d
Hard wheat whole starch	24.11±0.20e	27.9±0.26^f^	59.5±0.60c	65.88±0.06e	69.9±0.04d	10.4±0.57c	9.35±0.21f
Soft wheat A granules	28.19±0.32h	27.16±0.12^e^	59.74±0.12c	67.85±0.05f	71.57±0.04g	11.83±0.15d	9.00±0.2e
Hard wheat A granules	26.4±0.26g	28.06±0.03^f^	56.98±0.14b	62.08±0.07c	70.53±0.03f	13.55±0.16e	9.9±0.0818g
Soft wheat B granules	18.36±0.15d	23.94±0.11^c^	59.2±0.45c	62.33±0.10d	65.31±0.08a	6.11±0.37a	5.21±0.0360a
Hard wheat B granules	16±0.75c	27.06±0.14^e^	63.93±0.46d	68.38±0.08g	70.16±0.03e	6.23±0.44a	6.27±0.105b
Soft wheat C granules	14.5±0.17b	20.79±0.11^a^	59.37±0.21c	61.35±0.10b	67.69±0.04c	8.32±0.23b	6.27±0.065b
Hard wheat C granules	11.03±0.11a	22.63±0.24^b^	63.51±0.09d	69.94±0.03h	73.86±0.04h	10.35±0.07c	7.08±0.062c

All values represent the mean of three replicates.

Values are means ± standard error

Values followed by same letter in same row are not significantly different at p<0.05.

### Starch granules composition

Starch granule composition was calculated from elution peaks of gel permeation chromatography [S2 Fig]. Area under the first peak showed amylopectin content and under the second peak indicated amylose content. Soft wheat granules had higher amylose content than hard wheat granules. Between granules amylose content decreased in the order of A> B> C. Similar order was obtained for %age crystallinity. Previous observations indicated non-significant differences in hard and soft wheat total starch [20]. We observed significantly higher amylose content in soft wheat total starch. Amylose content of A granules was significantly higher than total, B- and C-type granules as reported previously [29,30]. Previous observations have indicated that the differences in crystallinity in different starches are due to differences in proportions of amylose, short side-chain and long side-chain amylopectin [31]. Location of the amylose in starch granules is under debate. Arragement of amylopectin glucan chains make up the crystalline region while branching point align to make up amorphous region of the granules [32]. High amylose granules have reported to exhibit low crystallinity [33]. But observed order of crystallinity by XRD diffraction in our study is A> B> C. It indicates that other factors play important role in determining crystallinity of starch granules.

### Thermal properties of starch granules

Transmittance of the starch granules (S1 Table) paste after boiling showed that A-granules of soft as well hard wheat formed clear paste. However hard wheat A granules had more clarity than soft wheat. Other granules like B- and C- formed fuzzy paste. If ever-industrial separation of starch granules becomes possible, A-granules from hard wheat would have better potential for biodegradable film formation due to more clear paste formation by them.

Thermal properties of the different starch granules as determined by DSC indicated that gelatinization enthalpy (ΔH) of different granules decreased in the order of A>B>C. ΔH has been related to degree of crystallinity [23]. This results is in agreement with the percentage crystallinity. ΔH of A-granules was even higher than total starch granules. ΔH of hard wheat starch granules was higher than soft wheat granules. Hard wheat A-granules exhibited lower onset gelatinization temperature (To) and peak gelatinization temperature (Tp) compared to soft wheat A-granules. Hard wheat C-granules with lowest amylose content had high To and Tp with medium ΔT indicating better stability [20].

### Reconstitution of starch granules and their thermal properties

**Fig 5:**
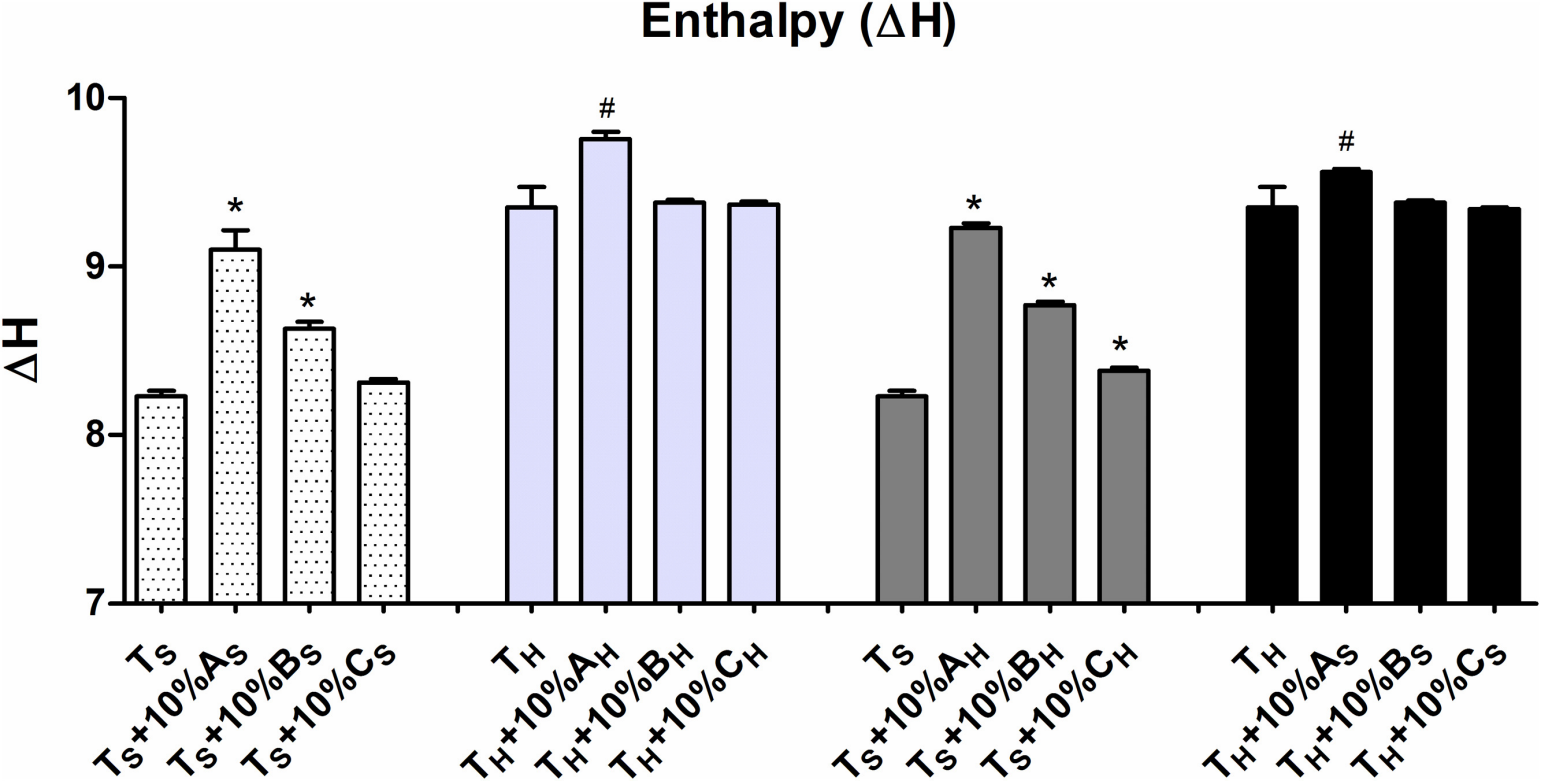
Comparative analysis of transition temperature enthalpy (ΔH) after 10% reconstitution (* and # significantly different at p value 0.05).

Reconstitution of soft wheat total starch with 10% of its own individual A-, B- and C- granules indicated significant changes in ΔH (Fig 5) and ΔT (S3 Fig). Highest increase in ΔH and ΔT was observed with addition of 10% A-granules. Significant increase was also observed after addition of B-granules with non-significant changes with C-granules. Similar reconstitution experiment with hard wheat increased ΔH and ΔT only in case of A-granules with non-significant change after B- and C-granules addition. When different types of hard wheat granules were added to opposite textured soft wheat total starch, highest increase in ΔH and ΔT values was observed after addition of A-granules. In opposite case in which different type of soft wheat granules were added to hard wheat total starch no change was observed in ΔT values but significant increase in ΔH was observed after addition of A-granules. Our results suggested that A-granules of hard wheat had highest effect on gelatinization properties of starch. These results suggest that starch granules properties can be modified by reconstitution. A-granules contribute 70% of starch volume. Additional 10% increase changed its properties significantly. Thus starches from soft wheat can be utilized similarly as hard wheat after substitution with A-granules from hard wheat. Further 10% addition of soft wheat starch granules to hard wheat will not change its technological properties.

### Correlation Analysis

**Fig 6:**
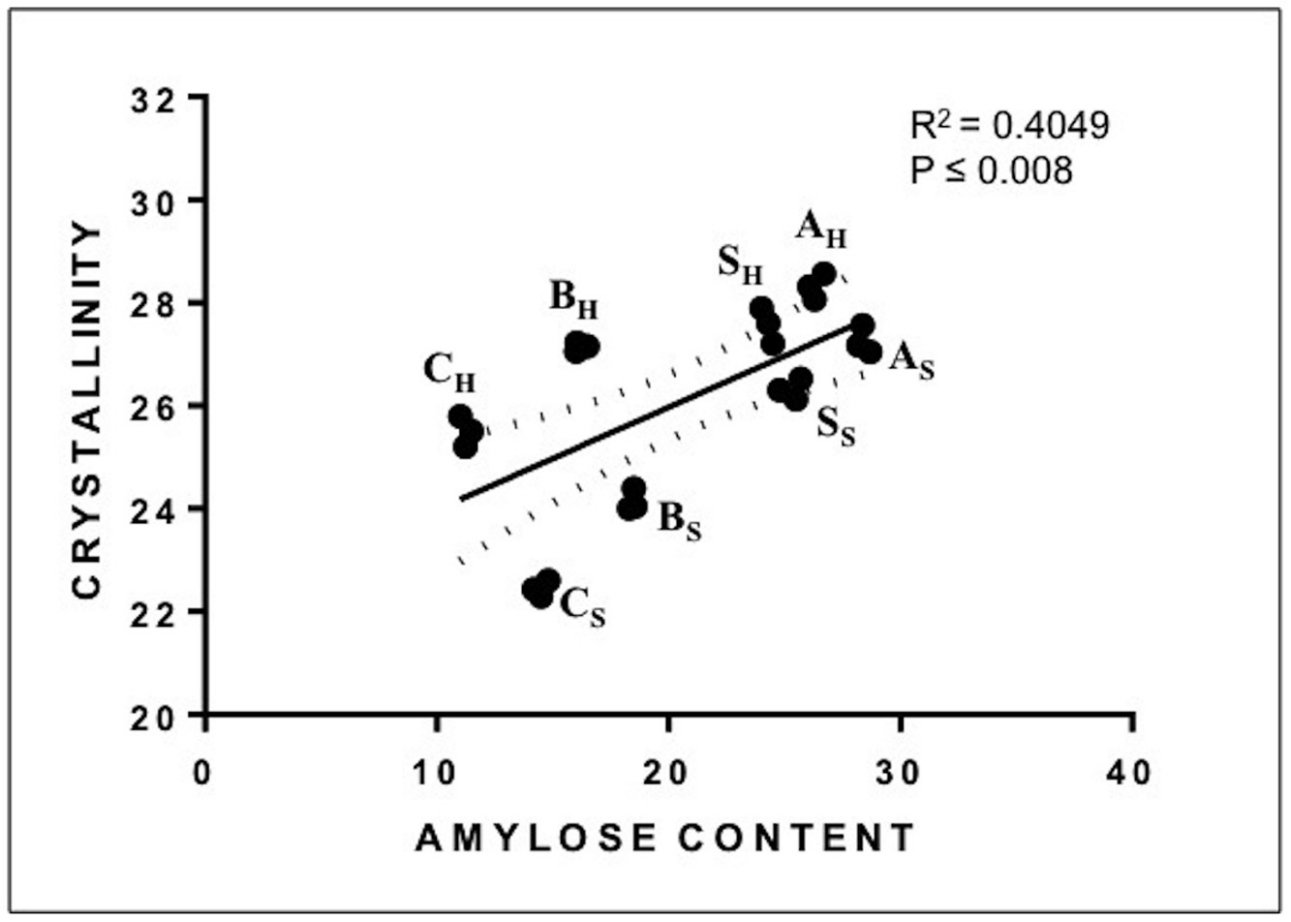
A graph showing positive correlation (p≤ 0.008) between amylose content and crystallinity.

Correlation analysis of crystallinity with amylose content has been shown in Fig 6. It indicated a positive correlation between crystallinity and amylose content between different types of starch granules, being highest in A-granules and lowest in C-granules. Within same type of granules from hard and soft wheat a negative correlation between crystallinity and amylose content was observed. Crystallinity plays a critical role in starch granules architecture and physiochemical characteristics [32,34,35,36,37]. The amylose is important component influencing various physiochemical properties of starch such as turbidity, syneresis, freeze-thaw stability, pasting, gelatinization and retrogradation properties [38]. In general a negative relation has been reported between amylose content and gelatinization [39]. Bigger amylopectin molecules provide higher viscosifying power and lower gelatinization temperature [40] compare to amylose. High amylose content starches retrograde easily and therefore have decreased recrystallization [41]. High amylose also stabilized the starch paste because it suppresses the swelling of starch granules [42]. Overall starch granules with high and low amylose content can have different applications.

Isolated amylose molecules showed crystalline structure with digestion resistance [43]. Digestion resistance starch reported to have health promoting effects like prevention of colon cancer and improvement of colon health [44,45]. A-granules from soft wheat showed highest amylose content or resistant starch. Amylose films have been reported to be crystalline while amylopectin films were amorphous [43]. Our results suggested that if ever separation of starch granules become possible, bigger A-granules with high amylose content and crystallinity may have good film forming, gelling and controlled digestion abilities [46]. A-granules from hard wheat with higher crystallinity, enthalpy and lower amylose content than soft wheat might be even better for the suggested usages. Smaller C-granules with low amylose content, low crystallinity and high viscosity might be more suitable for texturing, stabilizing, encapsulation and emulsification purposes [47]. C-granules of soft wheat with lower crystallinity, enthalpy and higher amylose content than hard wheat might be better for such purposes. Detailed examination of different properties is required for verification of our results. If verified our observations may encourage the breeders to develop wheat lines with single type of starch granules or higher abundance of particular type of starch granules.

### Substitution analysis of starch molecules

Substitution analysis of different starch granules as carried out by GC/MS. Table 2 represents the summary of the glucose recovered after GC/MS analysis of derivatized (partially methylated alditol acetates) monosaccharaides. Three abundant peaks observed belong to T-GlcP (1, 5-Di-O-aetyl-1-deuterio-2, 3, 4, 6- tetra-O- methyl-D-glucitol at 30.164 min), 4-GlcP (1, 4, 5-Tri-O-acetyl-1-deuterio- 2, 3, 6-tri-O-methyl-D-glucitol at 32.635 min) and 4, 6-GlcP (4,6-linked-D-glucopyranosyl residues at 34.988min) (S4 Fig). Substitution of starch granules was determined from the area under these peaks. Order of substitution of starch in various granules was A-granules > B-granules > C-granules (Table 2). Among soft and hard wheat, differences in the substitution pattern were identified as inferred from glucose (4,6- Glc) recovered (Table 2). These differences were observed in A-granules but not in B- and C-granules. A- granules of hard wheat were more substituted than soft wheat. The distribution of substituents in amylopectin represents the substitution pattern in the polymer back-bone [48]. This analysis provides a way to identify hydroxyl group of monosaccharide involved in glycosidic linkage. Degree of substitution relates to number of branches available in starch molecules. Branch length and their number has been related to degree of crystallinity. More amounts of branches and branch length will make bigger amylopectin molecules with increased intra- and inter-molecular bonding and higher crystallinity [7]. Due to high substitution of hard wheat granules they have high crystallinity compare to soft wheat. These observations are in congruence with birefringence, DSC and Powder XRD results.

**Table 2:** Summary of the glucose recovered after modification for substitution analysis.

Type of linkage	Soft wheat whole starch	Hard wheat whole starch	Soft wheat A granules	Hard wheat A granules	Soft wheat B granules	Hard wheat B granules	Soft wheat C granules	Hard wheat C granules
T- Glc	8.1±0.20^d^	6.7±0.36^c^	8.8±0.45^e^	8.1±0.30^d^	6.5±0.49^c^	5.6±0.25^ab^	5.2±0.19^a^	5.8±0.19^b^
4-Glc	85.7±0.35^c^	84.4±0.15^b^	84.4±0.30^b^	81.8±0.12^a^	87.9±0.83^d^	88.2±0.32^d^	89.4±0.32^e^	88.3±0.39^d^
4,6-Glc	5.9±0.25^ab^	8.8±0.38^d^	6.7±0.40^c^	9.9±0.41^e^	5.5±0.35^ab^	6.1±0.17^b^	5.4±0.18^a^	5.8±0.25^ab^

All values represent the mean of three replicates.

Values are means ± standard error

Values followed by same letter in same column are not significantly different at p<0.05.

## Conclusions

Differences were observed in structure and physiochemical properties between A-, B- and C-type granules isolated from hard and soft wheat. A-granules from hard wheat showed higher degree of substitution, crystallinity, enthalpy and lower amylose content. C-granules from soft wheat showed low crystallinity and higher amylose content. This study can form the basis for further comprehensive characterization of starch granules from soft and hard wheat.

## Supporting Information

S1 Fig Light microscopic images (10X) of starch granules. T_S_, A_S_, B_S_ and C_S_ represents soft wheat total starch, A granules, B granules and C granules respectively. T_H_, A_H_, B_H_ and C_H_ represents hard wheat total starch, A granules, B granules and C granules respectively.
(TIF)

S2 Fig Gel permeation chromatography elution curves of starch granules. (TIF)

S3 Fig Comparative analysis of transition temperature (ΔT) after 10% reconstitution (* and # significantly different at p value 0.05). (TIFF)

S4 Fig Characteristics GC/MS peaks obtained after matching with CCRC database for terminal linkages in starch. (TIF)

S1 Table Light transmittance of starch granules paste. (PDF)
