# Supplementary figures and images for: Soft and Hard Textured Wheat Differ in Starch Properties as Indicated by Trimodal Distribution, Morphology, Thermal and Crystalline Properties
Source: PLoS One. 2016 Jan 29;11(1):e0147622. doi: 10.1371/journal.pone.0147622 (PMC4732664; doi:10.1371/journal.pone.0147622)

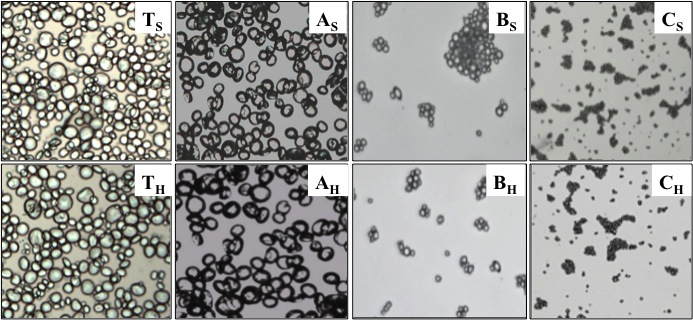

Supplement: S1 Fig — TS, AS, BS and CS represents soft wheat total starch, A granules, B granules and C granules respectively. TH, AH, BH and CH represents hard wheat total starch, A granules, B granules and C granules respectively. (TIF) [file pone.0147622.s001.tif]

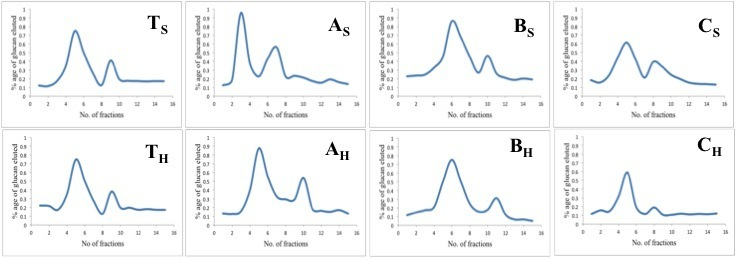

Supplement: S2 Fig — (TIF) [file pone.0147622.s002.tif]

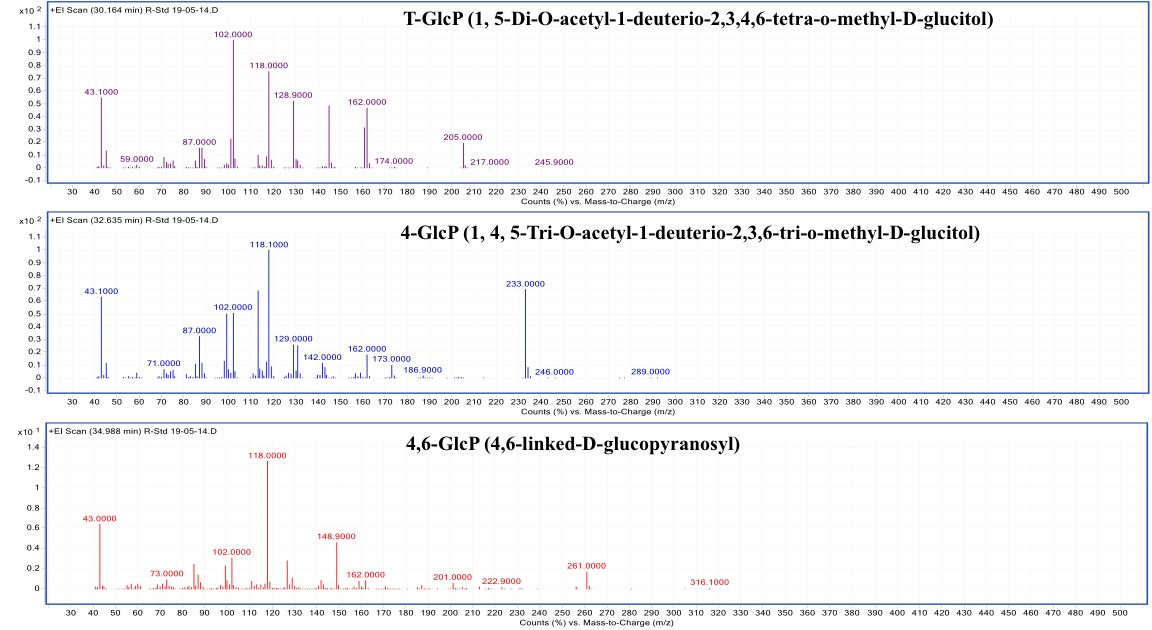

Supplement: S4 Fig — (TIF) [file pone.0147622.s004.tif]
