# Supplementary material for: Soft and Hard Textured Wheat Differ in Starch Properties as Indicated by Trimodal Distribution, Morphology, Thermal and Crystalline Properties
Source: PLoS One. 2016 Jan 29;11(1):e0147622. doi: 10.1371/journal.pone.0147622 (PMC4732664; doi:10.1371/journal.pone.0147622)
Supplement: S1 Table — (PDF) [file pone.0147622.s005.pdf]

**Starch granules (1%)**	**Transmittance (% at 650 nm)**
S1	77.82
A1	82.8832
B1	70.7402
C1	66.4252
S2	80.8226
A2	86.0465
B2	75.5903
C2	68.6065

All values represent the mean of three replicates.
